# Severe Hypothyroidism Presenting With Rhabdomyolysis in a Young Patient

**DOI:** 10.7759/cureus.13993

**Published:** 2021-03-19

**Authors:** Imran Janjua, Tabinda Bashir, Muhammad Zaka ul Haq, Muhammad Fahad Arshad, Muhammad Sharif

**Affiliations:** 1 Internal Medicine, Hamad Medical Corporation, Doha, QAT; 2 Family Medicine, Primary Health Care Corporation, Doha, QAT; 3 Department of Oncology and Metabolism, University of Sheffield, Sheffield, GBR

**Keywords:** rhabdomyolysis, hypothyroidism, creatinine kinase. acute kidney injury, myoglobulin

## Abstract

Rhabdomyolysis is an uncommon but potentially life-threatening medical condition. The acute muscle breakdown leads to the release of toxic muscle contents which can damage the kidneys and can cause the development of acute kidney injury (AKI) and fatal electrolyte imbalances associated with high morbidity and mortality. There are a variety of causes including exposure to extremely hot weather, toxins, trauma, certain drugs, and rarely endocrine disorders in particular thyroid dysfunction. It is more common among a certain group of people, for example, enthusiastic athletes, physical laborers, military and police personnel working in hot and humid weather. Moreover, it is also seen in patients using certain medications, and in the elderly following a fall and prolonged laying on the floor. The majority of the patients develop acute kidney failure and treatment with intravenous hydration and the underlying cause remains the mainstay of management.

Our case demonstrates the rare occurrence of AKI induced by rhabdomyolysis in patients with severe hypothyroidism. A 36-years-old male presented with generalized body pains, arthralgias, weight gain, and ankle edema of three months duration. On investigations, he was found to have severe hypothyroidism, AKI along with raised creatinine kinase (CK) and myoglobin indicating severe muscle damage. He was treated with intravenous (IV) fluids and oral levothyroxine in accordance with endocrine team advice.

This case highlights the significance of investigating acute rhabdomyolysis with thyroid function tests if no other cause is apparent from history like hyperthermia/drugs/toxins as in our case. Timely diagnosis and treatment of underlying pathology improve patient outcomes.

## Introduction

Rhabdomyolysis is skeletal muscle injury leading to the release of potentially toxic muscle fiber contents into the blood circulation [1]. The muscle necrosis may be due to direct or indirect injury and the resultant consequences may range from a mild muscle enzyme abnormality to life-threatening complications. The most common causes are trauma, exposure to extremely hot weather, myopathy, infections, electrolyte abnormalities, drugs such as statins and toxins [2]. Very rarely it is associated with endocrine causes including severe hypothyroidism as in our case.

Here, we present a case of a young patient who presented with rhabdomyolysis leading to acute kidney injury (AKI) and was found to have severe hypothyroidism.

## Case presentation

A 36-years-old Indian male, working as a hydraulic engineer at the airport, was admitted with symptoms of generalized body aches, ankle edema, and arthralgias for three months. There was no history of fever, rash, joints swelling or stiffness, shortness of breath, and chest pain. On direct questioning, he reported weight gain and hair loss. He denied any symptoms pertaining to respiratory, genitourinary, or gastrointestinal systems.

He was living alone, used to smoke four cigarettes/day until one year ago when he managed to quit successfully. He was consuming occasional alcohol but only in small amounts at weekends only. He had no known medical illnesses or allergies. There was no history of herbal or over-the-counter medicine use.

On admission, he was afebrile with a pulse of 75 beats/min, blood pressure of 150/95 mm of Hg, and respiratory rate of 18/min. He was overweight with a BMI of 28 kg/m^2^. General physical examination revealed mild non-pitting leg oedema. There was no joint swelling, skin rash, oral or genital ulcers. His systemic examination including cardiovascular, respiratory, gastrointestinal, and central nervous systems was unremarkable.

Baseline investigations showed (Table 1) AKI with significantly elevated levels of creatinine kinase (CK) and myoglobin. In view of his weight gain, a thyroid profile was done which was suggestive of severe hypothyroidism (thyroid-stimulating hormone [TSH] >100 mIU/L (normal 0.3-4.20 miU/L) and free T4 <0.5 pmol/L (normal 11.0 - 23.3 pmol/L). The anti-thyroid peroxidase antibody (anti-TPO Ab) was 212 IU/ml (reference range (0-34IU/L). Interestingly, he was noted to have low early morning cortisol levels and response to adrenocorticotrophin hormone (ACTH) was also subnormal (baseline 254 nmol/L, at 30 min 348 nmol/L and at 60 min 288 nmol/L) suggesting underlying adrenal insufficiency (AI). The baseline ACTH was 67.7 pg/ml (reference range (7.2-63.3 pg/ml), suggestive of primary AI.

**Table 1:** Laboratory Investigations CK: creatinine kinase, ALT: alanine transaminase, AST: aspartate aminotransferase, TSH: thyroid-simulating hormone, CRP: C-reactive protein.

	Day 1	Day 5	At discharge	4 weeks
Haemoglobin (g/dl) (normal 13-17)	12.7	12.3	11.0	11.0
White cell count (normal 4-10)	6.8	8.0	8.1	8.0
Platelets (normal 150-400)	280	272	238	231
Creatinine (µmol/L) (normal 68-106)	123	157	113	122
Urea (mmol/L) (normal 2.8-8.1)	4.6	3.7	3.7	5.5
Sodium (mmol/L) (136-145)	136	136	140	NA
Potassium (mmol/L) (3.5-5.1)	4.3	4.0	4.7	NA
CK (U/L) (normal 39-308)	10891	5541	3852	554
ALT (U/L) (normal 0-41)	4	51	40	26
AST (U/L) (normal 0-40)	114	110	94	74
Myoglobin (ng/ml) (normal 28-72)	714	474	200	NA
TSH (mIU/L) (normal 0.3-4.20)	>100	>100	-	9.23
Free T4 (pmol/L) (normal 11-23.3)	<0.5	3.8	5.7	11.4
Total cholesterol (mmol/L) (normal <5.2)	9.0	NA	NA	5.5
Triglyceride (mmol/L) (normal <1.7)	9.2	NA	NA	4.8
CRP (mg/L) (normal <5.0)	<0.3	<0.30	0.4	NA
HbA1c (%) (normal <5.7)	5.9%	NA	5.6%	NA

Other investigations including antinuclear (ANA), anti-cyclic citrullinated peptide (CCP), antineutrophil cytoplasmic (ANCA), anti-Jo, anti-La, anti-liver-kidney microsomal (LKM), and anti-mitochondrial (AMA) antibodies were negative with normal complement levels. Hepatitis serology and ultrasound examination of the hepatobiliary area were also normal as well as baseline electrocardiogram (ECG) and echocardiogram.

He was treated with intravenous fluids and oral Levothyroxine after consulting the local endocrine team. He was also commenced on steroids for AI (hydrocortisone 10 mg in the morning and 5 mg in the evening). He responded well to the above treatment regimen and discharged on day 8. He was followed up in the endocrine clinic and repeat blood after six weeks showed marked improvement. His laboratory parameters with reference ranges are summarized in the table.

## Discussion

Rhabdomyolysis very often presents with the classical triad of muscle pain, weakness, and dark urine [3-6], however, many patients may not report any muscular symptoms.

The characteristic feature of rhabdomyolysis is the rise of muscle enzymes including CK, aldolase, lactic dehydrogenase (LDH), and transaminases. But the distinctive attribute that has great diagnostic utility is a rise of CK, at least, five times the upper normal value [7]. CK levels start rising within 12 hours of insult to muscles and peak in approximately 72 hours. If there is no ongoing muscle injury, CK levels start to decline at day 5. CK has a serum half-life of about 1.5 days and declines at a relatively constant rate of about 40% to 50% of the previous day's value [8,9]. The damaged muscle fibers also lead to the release of myoglobin, a heme-containing protein, which in higher concentrations is highly nephrotoxic and can result in renal tubular obstruction, ischemia, and AKI [10,11]. In general myoglobin concentrations do not correlate with CK in rhabdomyolysis. Its level rises ahead of CK in blood and declines while CK level may still be having an upward trend [9]. This is likely due to the factors such as short half-life, less protein binding ability, and rapid excretion through kidneys.

Other abnormalities that can result from rhabdomyolysis include electrolyte disturbances, AKI, liver dysfunction, and even disseminated intravascular coagulation in extreme cases. The most common electrolyte abnormalities are hyperkalemia and hyperphosphatemia as potassium and phosphate are released from damaged muscle fibers. The severity of the hyperkalemia and hyperphosphatemia is linked to low effective circulatory volume, thus associated with AKI.

On the other hand, hypothyroidism usually presents with fatigue, cold intolerance, weight gain, constipation, dry skin, myalgia, and menstrual irregularities. Patients could be noted to have a goiter (especially with iodine deficiency or Hashimoto's thyroiditis), bradycardia, diastolic hypertension, and a delayed relaxation phase of the deep tendon reflexes. Patients with chronic autoimmune thyroiditis may have elevated levels of thyroid peroxidase (TPO) antibodies. Other abnormalities may include hypercholesterolemia, macrocytic anemia, elevated CK, and hyponatremia [3]. In addition, hypothyroidism is well known to be associated with liver dysfunction especially elevated aspartate aminotransferase (AST) in relation to alanine transaminase (ALT). The elevation of AST can also be contributed by muscle breakdown.

There have only been a few cases reported in the literature so far where hypothyroidism is clearly linked with rhabdomyolysis like our case [2,12,13]. It has been postulated that hypothyroidism can also result in muscle necrosis that leads to the release of intracellular muscle constituents into the circulation resulting in a marked elevation of CK levels, which is typical for this condition. In addition, rhabdomyolysis in the wake of hypothyroidism is more common and reported in the literature with concomitant statin use or associated exposure to extremely hot weather. 

Our patient had a CK level of more than 50 times the upper limit along with the clinical symptoms of generalized body aches, ankle edema, weight gain, hair loss, and arthralgias. His laboratory examination revealed a TSH of >100 mIU/L with low FT4 and myoglobin of 714 ng/ml (normal 28-72 ng/L). He did not have any other risk factors for the development of rhabdomyolysis and had negative workup for infections, inflammatory, or autoimmune etiology. He also had negative hepatitis serology and a normal ultrasound of the hepatobiliary area. These findings led to the diagnosis of hypothyroidism-induced rhabdomyolysis.

The major aspects of the treatment of patients with rhabdomyolysis include; recognition and management of fluid and electrolyte abnormalities, identification of the specific causes and their management (including discontinuation of drugs or other toxins that may be a contributing factor), and prompt recognition, evaluation, and treatment of compartment syndrome in patients whom it is present. AKI is one of the most common complications with the reported frequency ranging from 15% to over 50%, and hence, early and aggressive fluid resuscitation is key to prevent it [14].

The type of fluid and rate of repletion is unclear, however, initial fluid resuscitation with isotonic saline at the rate of 100-200 ml/hour is recommended to maintain renal perfusion, thereby minimizing ischemic injury. All patients should be initially treated with vigorous fluid repletion until it is clear from serial laboratory values that the plasma CK level is stable and not increasing. Patients who have a stable plasma CK level <5000 unit/L do not warrant intravenous fluid, since studies have shown that the risk of AKI is low among such patients [9].

In severe hypothyroidism, measurement of TSH and free T4 should be carried out every four to six weeks to check the response of treatment and the dose should be adjusted by 25-50 mcg/day to achieve the therapeutic goal. Our patient was also noted to have low early morning cortisol levels and his response to ACTH was also subnormal. Hence, he received steroids in addition to intravenous fluids and levothyroxine. It is not uncommon to find other autoimmune conditions such as Addison's disease, vitiligo, pernicious anemia, type 1 diabetes mellitus with autoimmune hypothyroidism.

We suggest that hypothyroidism should be considered as one of the differential diagnoses in patients with rhabdomyolysis. American Thyroid Association clinical guideline recommends screening patients for hypothyroidism who have an increase in serum concentration of either CK or LDH or both for at least two weeks [15]. Our case emphasizes the significance of screening for hypothyroidism in patients with rhabdomyolysis and symptoms of hypothyroidism in the absence of other causes.

## Conclusions

Rhabdomyolysis results from skeletal muscle damage and can cause several complications including electrolyte disturbances, renal failure, liver dysfunction, and occasionally disseminated intravascular coagulation. It can be induced by a variety of causes including rare causes like severe uncontrolled hypothyroidism. Therefore, clinicians should be aware of the potential risk of hypothyroidism-induced rhabdomyolysis and consider the possibility of underlying hypothyroidism in patients presenting with rhabdomyolysis in whom there is no apparent cause identified on the basis of history and investigations. In addition, concomitant use of certain medications such as statins or trimethoprim-sulfamethoxazole can also predispose hypothyroid patients to rhabdomyolysis.

The management of rhabdomyolysis due to severe hypothyroidism relies on general principles including fluid replacement and electrolyte correction, in addition to the treatment of thyroid dysfunction. Endocrine team input should be sought especially in complicated cases, and in those refractory to treatment. Early recognition and prompt treatment have ensured full recovery as in our case.
